# Case Report: Acute acalculous cholecystitis due to *Salmonella Typhi*: a case of conservative treatment failure and review of the literature

**DOI:** 10.3389/fsurg.2026.1787846

**Published:** 2026-03-26

**Authors:** Alessio Pasquale, Marco Brolese, Laura Marinelli, Stefano Valcanover, Stefano Marcucci, Paolo Beltempo, Cristina Prezzi, Marco Frisini, Alberto Brolese

**Affiliations:** 1 Department of General Surgery, Hepato-Pancreato-Biliary (HPB) Unit—Azienda Sanitaria Universitaria Integrata del Trentino (Asuit), Trento, Italy; 2 Department of Surgery, Oncology and Gastroenterology, Hepatobiliary Surgery and Liver Transplantation, Padova University, Padova, Italy

**Keywords:** acute acalculous cholecystitis, conservative treatment failure, laparoscopic cholecystectomy, *Salmonella Typhi*, typhoid fever

## Abstract

**Background:**

Acute acalculous cholecystitis (AAC) is an uncommon but potentially life-threatening inflammation of the gallbladder in the absence of gallstones, accounting for 5–10% of all cases of acute cholecystitis. While most cases occur in critically ill or postoperative patients, infectious etiologies—particularly *Salmonella Typhi*—are increasingly recognized. Typhoid-related AAC is well described in children from endemic regions but remains rare in adults, with limited evidence to guide management.

**Case presentation:**

We report the case of a 67-year-old man presenting with fever, chills, and epigastric pain following a self-limited diarrheal illness. Laboratory findings showed marked leukocytosis, elevated transaminases, and hyper-bilirubinemia with mild pain in the right upper quadrant. Blood cultures showed an infection of *Salmonella Typhi* sensitive to ceftriaxone. Despite 72 h of targeted antibiotic therapy, the patient remained febrile with rising inflammatory markers. Laparoscopic cholecystectomy was performed, revealing a gangrenous, necrotic gallbladder. Histopathology confirmed AAC with wall necrosis and abscesses. The postoperative course was uneventful.

**Conclusion:**

This case highlights the importance of recognizing *Salmonella*-associated AAC as a rare but severe complication of typhoid infection in adults. While initial conservative therapy is reasonable, persistent symptoms or lack of improvement after 48–72 h should prompt early surgical intervention to prevent gallbladder necrosis, perforation, and sepsis.

## Introduction

Acute cholecystitis is characterised by right upper quadrant pain, fever, and leucocytosis caused by inflammation of the gallbladder. It accounts for 3%–10% of all cases of abdominal pain and typically occurs in patients with gallstones (acute calculous cholecystitis, ACC). A less common presentation is acute acalculous cholecystitis (AAC), which comprises 5%–10% of all cases (1, 2).

AAC is an acute necro-inflammatory condition of the gallbladder that develops in the absence of cystic duct obstruction (3). It is believed that bile stasis, together with gallbladder wall oedema and reduced blood supply, contributes to ischaemia and subsequent gallbladder injury (2, 4, 5).

The pathogenesis of AAC is multifactorial and has been associated with severe burns, trauma, critical illness, cardiovascular surgery, total parenteral nutrition, and systemic infections, reflecting in most cases the progression towards multi-organ dysfunction (3). A particularly important subset of cases arises from direct microbial invasion of the gallbladder. Numerous pathogens have been shown to precipitate AAC through epithelial infiltration and local inflammation, including *Escherichia coli, Enterococcus* spp., *Klebsiella *spp., *Pseudomonas *spp., *Proteus* spp., *Bacteroides* spp, *Salmonella *spp., *cytomegalovirus, Cryptosporidium* spp., *Isospora belli, Sarcocystis* spp., *Cyclospora cayetanensis, Enterocytozoon bieneusi, Histoplasma capsulatum, and Mycobacterium tuberculosis* (6).

Given its rapid onset and progression, timely diagnosis is critical, as acute AAC can carry a mortality rate of up to 65%; however, early recognition and prompt intervention can reduce mortality to approximately 7% (2).

AAC secondary to Salmonella infection is more prevalent in children living in endemic regions (1, 7), whereas it remains an uncommon diagnosis in adults, particularly in developed countries (1). In paediatric populations, Salmonella-associated AAC accounts for approximately 50%–70% of cases, compared with only 2%–15% in adults. This condition is also associated with substantially higher morbidity and mortality, especially in children (7). Nevertheless, no specific guidelines exist regarding the optimal management of AAC caused by *Salmonella Typhi*.

## Case presentation

We report the case of a 67-year-old man who developed acute acalculous cholecystitis (AAC) in association with Salmonella typhi gastroenteritis at our institution.

The patient was admitted to the Gastroenterology Department presenting with fever, chills, and epigastric pain. One week prior, he experienced three days of watery diarrhea, with 10–15 episodes per day, which resolved spontaneously. His medical history included ischaemic heart disease, with no other relevant comorbidities.

On admission, laboratory investigations revealed elevated inflammatory markers, including significant leukocytosis (WBC 19.6 × 10^9^ /L), markedly increased C-reactive protein (CRP) (69.5 mg/L), and procalcitonin (PCT) (26.4 ng/mL). Liver function tests showed elevated bilirubin levels (total 4.3 mg/dL, direct 2.2 mg/dL) and significantly increased transaminases (AST 622 U/L, ALT 728 U/L) and cholestatic enzymes (ALP 236 U/L, GGT 265 U/L). The patient also reported mild right upper quadrant abdominal pain.

Following the isolation of *Salmonella Typhi* from blood cultures taken on admission, which was fully sensitive to ceftriaxone, intravenous ceftriaxone (2 g/day) was promptly initiated.

**Figure 1:**
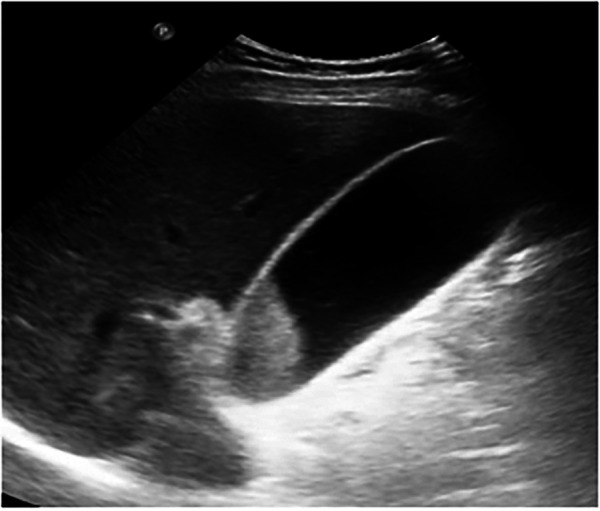
Ultrasound: the gallbladder appears distended with coarse biliary sludge and well-defined walls, without evidence of gallstones or biliary duct dilatation.

Initial abdominal ultrasound showed a distended gallbladder with well-defined walls and coarse biliary sludge, without pericholecystic fluid or dilation of the intra- or extrahepatic bile ducts (Figure 1).

In the first days after admission, laboratory tests revealed a significant increase in inflammatory markers: white blood cell count rose to 22 × 10^9^ /L, CRP to 155 mg/L, and PCT to 25 ng/mL. Liver function tests showed elevated total bilirubin (6.5 mg/dL) with an increased direct fraction (3.4 mg/dL), suggestive of cholestasis. To exclude biliary obstruction, an endoscopic ultrasound (EUS) was performed, confirming the presence of biliary sludge without dilation of the bile ducts.

Despite ongoing antibiotic therapy, no improvement was observed by day three: white blood cells decreased slightly to 15 × 10^9^ /L, bilirubin levels remained stable, CRP increased to 162 mg/L, and procalcitonin decreased to 6.85 ng/mL. Clinically, the patient continue to experience right upper quadrant pain and persistent fever, indicating an inadequate response to medical treatment.

**Figure 2:**
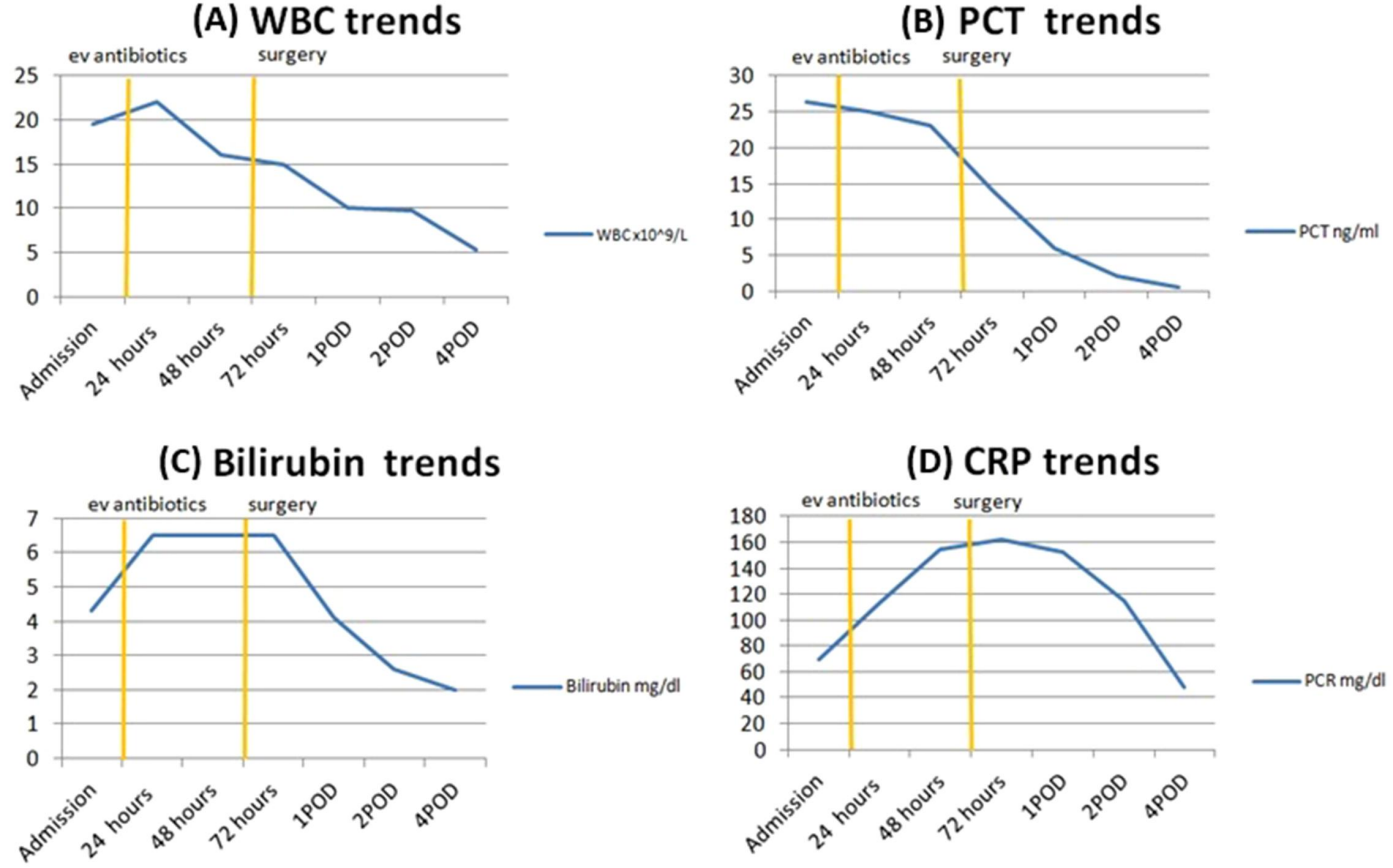
Trends of laboratory parameters during patient hospitalization. Each panel shows the time course of a specific laboratory marker: **(A)** White blood cell count (WBC, ×10^9^ /L), **(B)** Procalcitonin (PCT, ng/mL), **(C)** Bilirubin (mg/dL), and **(D)** C-reactive protein (CRP, mg/dL). Vertical lines indicate the start of empirical antibiotic therapy and the time of surgery. Time points correspond to admission, hours after admission, and postoperative days (POD).

Due to persistent fever and systemic inflammation despite 72 h of targeted antibiotic therapy, a multidisciplinary discussion involving surgeons and infectious disease specialists concluded that surgical intervention was indicated (Figure 2).

The patient subsequently underwent laparoscopic cholecystectomy. Intraoperative findings revealed a severely inflamed gallbladder with extensive gangrenous and necrotic changes. The procedure lasted 110 min, with an estimated blood loss of approximately 100 mL.

The postoperative course was uneventful. The patient resumed oral intake on postoperative day one, mobilised without difficulty, and was discharged home on postoperative day four. Histopathological examination confirmed AAC with extensive wall necrosis and intramural abscesses.

## Discussion

Salmonellae are motile, gram-negative bacilli that can be divided into two broad categories: typhoidal strains, such as *Salmonella* Typhi, which cause typhoid and enteric fever, and the broader group of non-typhoidal Salmonella responsible for gastroenteritis (8).

Although hepatobiliary involvement is uncommon, Salmonella infection may reach the gallbladder through haematogenous spread, lymphatic dissemination, or ascending migration from the gastrointestinal tract (6).

The first reported case of acute acalculous cholecystitis (AAC) secondary to Salmonella infection was described by Lothrop in 1915 (9). Experimental and histopathological studies have since demonstrated a distinct tropism of *Salmonella Typhi* for gallbladder epithelial cells, with high bacterial concentrations detected within both the lumen and gallbladder wall. This bacterial invasion, ultimately leading to epithelial injury and tissue destruction (6, 10). These findings provide a plausible pathophysiological explanation for AAC in the absence of gallstones.

Clinically, symptom onset may be insidious; however, progression to severe systemic manifestations such as sepsis, shock, or peritonitis due to gallbladder necrosis, gangrene, or perforation has been reported (11). Once AAC is diagnosed, prompt treatment is essential, as ongoing ischaemia may rapidly evolve into gangrene and perforation.

At present, there is no unanimous consensus regarding the optimal management of Salmonella-associated AAC. According to the Tokyo Guidelines 2018, early laparoscopic cholecystectomy—preferably within 72 h of symptom onset or during the same hospital admission—is recommended for patients with mild (grade I) and moderate (grade II) acute cholecystitis. In severe cases (grade III), gallbladder drainage, such as percutaneous cholecystostomy, is advised in haemodynamically unstable patients or in those unfit for general anaesthesia (12).

However, evidence derived from published case reports suggests that many patients with Salmonella-associated AAC, particularly those who are haemodynamically stable and without complications, may achieve favourable outcomes with conservative treatment consisting of intravenous fluids and targeted antibiotic therapy alone (4, 13, 14). Successful conservative management has also been described in patients presenting with concomitant severe conditions, including massive intestinal bleeding or other extraintestinal manifestations, provided that no signs of gallbladder gangrene or perforation were present (15–17). These observations support an initial non-operative approach in carefully selected patients under close clinical monitoring.

Nevertheless, conservative treatment is not universally effective. Several reports describe clinical deterioration despite appropriate antibiotic therapy, ultimately requiring surgical intervention (1, 18). In some cases, failure of medical management became evident within 36–72 h of treatment initiation (8). Furthermore, immediate surgical management—either open or laparoscopic cholecystectomy—has been employed as first-line therapy in the presence of clinical instability or complications such as empyema, gangrene, or perforation (19, 20). Cases of biliary peritonitis secondary to gallbladder perforation have also been documented, underscoring the potentially life-threatening nature of this condition (21). Comparable findings have been reported in additional adult cases in which rapid progression to gangrene or perforation occurred despite targeted antimicrobial therapy, necessitating definitive surgical treatment (22–25).

For critically ill patients with high perioperative risk, percutaneous cholecystostomy represents a valuable alternative, with most patients demonstrating clinical improvement within 48 h of the procedure (26, 27).

An additional consideration is the risk of chronic carriage. Approximately 3%–5% of individuals infected with Salmonella may become chronic carriers, with the gallbladder serving as a bacterial reservoir, regardless of the presence of gallstones (4). Chronic infection of the gallbladder mucosa has also been associated with an increased risk of gallbladder carcinoma (28, 29), highlighting the importance of appropriate follow-up in selected cases.

Overall, the available evidence—largely based on isolated case reports—indicates that Salmonella-associated AAC is a rare but potentially severe complication. While conservative therapy appears effective in clinically stable patients without complications, early surgical intervention should not be delayed in cases of clinical deterioration or suspected gangrene or perforation. Management should therefore be individualised, guided by disease severity and response to therapy.

The principal limitation of this report lies in its single-case nature, which inherently restricts the generalisability of the findings. As with all isolated case descriptions, the results should be interpreted with caution.

Cases of Salmonella-associated acute acalculous cholecystitis (AAC) reported in the literature are summarized in Table 1.

**Table 1:** Reported adult cases of Salmonella-associated AAC.

Author (Year)	Age/Sex	Symptoms	Pathogen (indetification	Imaging findings	Treatment	Histology
Syma Iqbal et al. 2018 (4)	60 F	Generalized abdominal pain, watery diarrhea, vomiting	Salmonella (microbiologically)	US: pericholecystic fluid, no gallstones, normal wall thickness	Azithromycin ×5 days	n/a
El Bachir Benjelloun et al. 2013 (21)	65 M	Abdominal pain, nausea, vomiting (48 h)	S. Paratyphi B (microbiologically)	US + CT: wall thickening, pericholecystic collection, no stones	Emergency open cholecystectomy	Gangrenous cholecystitis
Rajan et al., 2014 (1)	24 F	Fever, vomiting, epigastric pain, malaise, headache	S. Typhi (microbiologically)	US: wall thickening, pericholecystic fluid, probe tenderness	Laparoscopic cholecystectomy	Chronic inflammatory infiltrate
Lianos et al., 2019 (8)	32 M	Abdominal pain, diarrhea, vomiting, fever 39°C	Salmonella (microbiologically)	US: wall thickening, pericholecystic fluid, no biliary dilatation	Emergency open cholecystectomy	Severe acute cholecystitis
Li et al., 2020 (26)	Elderly M	Fever, vomiting, diarrhea	Salmonella Group B (microbiologically)	US + CT: patchy wall enhancement, acute cholecystitis	Percutaneous cholecystostomy	Improved
Zhao et al., 2020 (17)	90 M	Persistent diarrhea, abdominal pain, fever 39.4°C	Salmonella Group D (microbiologically)	US + CT: sludge, no stones; CT: acute cholecystitis + appendicitis	Laparoscopic cholecystectomy + appendectomy	Gangrenous cholecystitis
Ruiz-Rebollo et al., 2008 (13)	27 M	Abdominal pain, vomiting, diarrhea, fever 38°C	S. enteritidis (microbiologically)	US: thickened wall, pericholecystic fluid	Ciprofloxacin + metronidazole	n/a
Khan et al., 2009 (14)	31 M	Fever, abdominal pain, vomiting	Salmonella (suspected)	US: AAC	Piperacillin/tazobactam	n/a
Inian et al., 2006 (22)	21 F	High-grade fever, vomiting, diarrhea (2 days)	S. Typhi (serology)	US: wall thickening, pericholecystic fluid	Ciprofloxacin + cefotaxime + metronidazole	n/a
Goel et al., 2020 (23)	24 F	Fever, vomiting, malaise, distension	S. Typhi (microbiologically)	US/CT: gallbladder perforation	Cholecystectomy	n/a
Juma et al., 2019 (24)	41 F	Severe epigastric pain, fever, vomiting, loose stools	S. enterica (microbiologically, bile)	US/MRCP: thick wall, pericholecystic fluid	Cholecystectomy	Necrotic gallbladder
Beyazal Polat et al., 2013 (16)	24 F	Abdominal pain, nausea, fever, headache, cystitis	S. Typhi (serology)	US: wall thickening, minimal pericholecystic fluid	Ceftriaxone 2 g OD × 14 days	n/a
Gong et al., 2021 (15)	21 M	Fever, anorexia, diarrhea, intestinal bleeding	S. enterica (microbiologically)	CT: acute cholecystitis	Meropenem 1 g TID	n/a
Khatri et al., 2009 (25)	40 M	Epigastric pain	S. Typhi (microbiologically)	x-ray: intestinal obstruction, no perforation	Open cholecystectomy	Necrotic gallbladder

US, ultrasound; CT, computed tomography; MRCP, magnetic resonance cholangiopancreatography; OD, once daily; TID, three times daily; n/a, data not reported.

## Conclusion

Typhoidal infection is a well-established cause of AAC, but evidence regarding optimal management and prognosis remains limited. This case highlights the importance of early recognition of complicated disease and timely intervention to achieve favorable outcomes. Given the paucity of data on the most effective treatment strategies for typhoidal AAC, clinical judgment remains essential in guiding the decision for prompt surgical intervention.

## Patient’s perspective

From the patient's perspective, the sudden onset of symptoms and the uncertainty regarding the need for surgery were the most distressing aspects of the disease. Undergoing surgical intervention after initial medical management was perceived positively, as it led to symptom resolution and a favourable clinical outcome.

## Data Availability

The original contributions presented in the study are included in the article/Supplementary Material, further inquiries can be directed to the corresponding author.
